# Psychiatric Morbidity and Other Factors Affecting Treatment Adherence in Pulmonary Tuberculosis Patients

**DOI:** 10.1155/2013/489865

**Published:** 2013-04-15

**Authors:** Argiro Pachi, Dionisios Bratis, Georgios Moussas, Athanasios Tselebis

**Affiliations:** Psychiatric Department, Sotiria General Hospital of Chest Disease, Athens, Greece

## Abstract

As the overall prevalence of TB remains high among certain population groups, there is growing awareness of psychiatric comorbidity, especially depression and its role in the outcome of the disease. The paper attempts a holistic approach to the effects of psychiatric comorbidity to the natural history of tuberculosis. In order to investigate factors associated with medication nonadherence among patients suffering from tuberculosis, with emphasis on psychopathology as a major barrier to treatment adherence, we performed a systematic review of the literature on epidemiological data and past medical reviews from an historical perspective, followed by theoretical considerations upon the relationship between psychiatric disorders and tuberculosis. Studies reporting high prevalence rates of psychiatric comorbidity, especially depression, as well as specific psychological reactions and disease perceptions and reviews indicating psychiatric complications as adverse effects of anti-TB medication were included. In sum, data concerning factors affecting medication nonadherence among TB patients suggested that better management of comorbid conditions, especially depression, could improve the adherence rates, serving as a framework for the effective control of tuberculosis, but further studies are necessary to identify the optimal way to address such issues among these patients.

## 1. Introduction

Tuberculosis (TB) is a chronic infectious multisystemic disease caused by mycobacterium tuberculosis [1] and is one of the leading causes of mortality worldwide [2–4]. The World Health Organization (WHO) has estimated that 2 billion people, almost a third of the world's population, have latent TB [5, 6]. Every year about eight million people develop this disease, and some three million die of it, over 95% of these from developing countries [7, 8]. In 2005 the highest rates per capital were from Africa (28% of all TB cases), and half of all new cases were from six Asian countries, namely, Bangladesh, China, India, Indonesia, Pakistan, and the Philippines [9, 10].

Beginning in 1985, a resurgence [11–14] of TB was observed, primarily in certain groups, including the homeless [15], those who are HIV seropositive [16], individuals with a history of alcohol or drug abuse [17], and immigrants from a country in which TB is endemic [18]. 

Many recipients of psychiatric services possess one or more of these risk factors [19, 20], and, consequently, TB may be overrepresented in this population. Conversely, psychiatric illness may develop subsequent to TB infection, and mood disorders seem to be particularly common in TB patients compared with those with other medical diagnoses [21–24].

The World Health Organization's (WHO) goal of tuberculosis control remains elusive [25, 26], and this failure has been blamed on numerous factors such as inadequate emphasis on human dimension of tuberculosis control [27–29] which includes nonadherence with medication and results in multidrug resistance and therapeutic failure [30, 31].

The purpose of this study is to investigate factors associated with medication nonadherence among patients suffering from tuberculosis with emphasis on psychopathology as a major barrier to treatment adherence [32, 33]. 

The literature indicates that psychiatric comorbidity [34] before and after tuberculosis onset, psychological issues such as stigma [35], isolation [36], sense of social support [37], helplessness [38], and other psychological reactions to the disclosure of the diagnosis [39] as well as medication side-effects [40], all adversely affect the treatment adherence. 

On the other hand, studies report high prevalence rates of psychiatric comorbidity among patients with drug-resistant tuberculosis [40] and that prevalence of depression significantly correlates with severity and duration of the disease [41, 42]. 

The causal relationships between mental disorders and tuberculosis are complex. Severe mental disorders are associated with high risk of tuberculosis acquisition and transmission [43] and with poorer adherence to anti-TB treatment. Conversely, diagnosis with tuberculosis increases risk of psychiatric comorbidity.

Only by taking these observations together along with the fact that the prevalence of tuberculosis rises in specific groups [179] especially among individuals who are more likely to be psychologically distressed than the general population, such as homeless, immigrants, and HIV patients, does a complete picture emerge, and researchers conclude that in order to increase the cure rates of tuberculosis psychiatric, comorbidity must be firstly identified and treated [82].

## 2. Method 

### 2.1. Search Strategy

**Figure 1:**
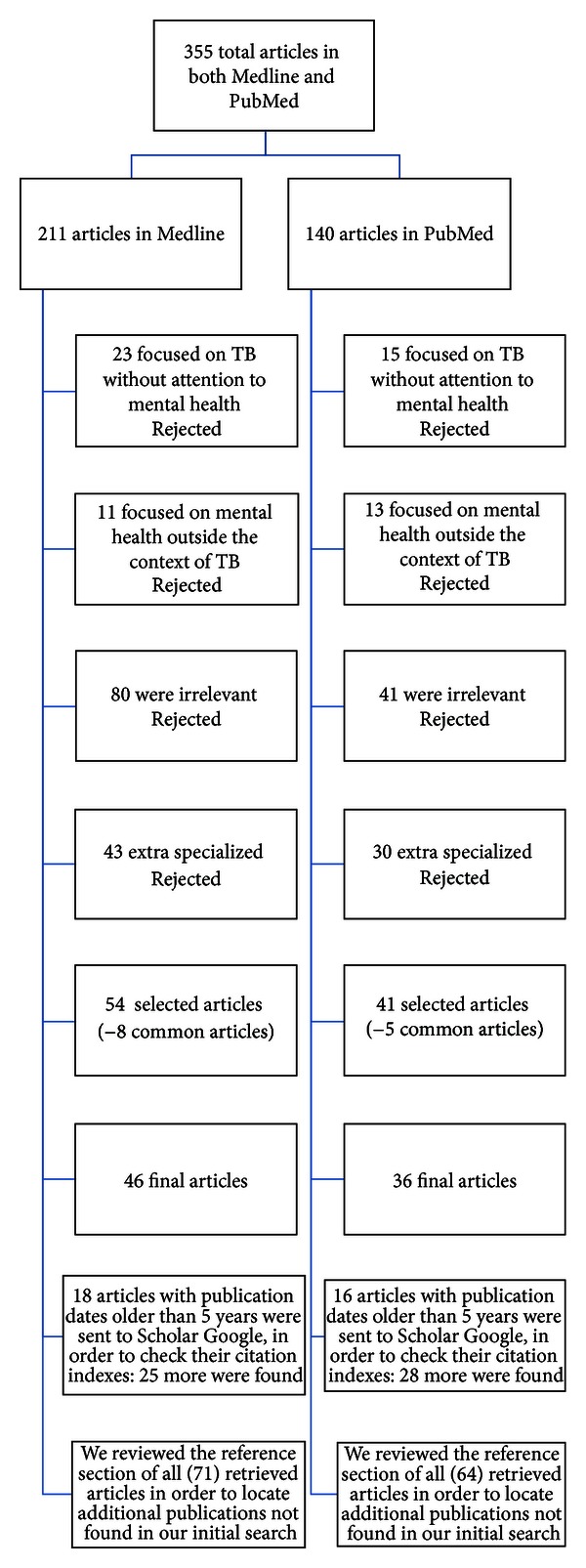
Consort diagram of the method.

Using the terms “tuberculosis and mental illness,” “anxiety and depression in tuberculosis,” “risk factors for nonadherence to tuberculosis treatment,” and “psychiatric and antitubercular drugs,” we searched Medline and using the terms “mental illness stigma and tuberculosis,” “mental illness primary care and tuberculosis,” and “tuberculosis non-adherence and psychosocial factors,” we searched PubMed. Articles were restricted to English, and publication dates were from 1950 to 2012. This yielded 355 articles. However, after excluding 38 articles that focused on TB without attention to mental health, 24 articles on mental health outside the context of TB and another 121 irrelevant and 73 extra specialized articles outside our primary focus, 94 articles were found, but 82 were kept for review, after excluding common articles. Then, 34 articles with publication dates older than 5 years were sent to Scholar Google in order to check their citation indexes. With this method, we located 53 additional articles with publication dates during the last 5 years. We reviewed the reference section of all (135) retrieved articles in order to locate additional publications not found in our initial search (Consort diagram—Figure 1).

We reviewed each article, noting date of publication, key results, and conclusions and then assigned the articles to different categories according to our primary focus.

Firstly, we reviewed the past medical literature, from an historical perspective. Then, we theorized upon issues regarding the complex interrelation and interaction between psychiatric disorders and tuberculosis, followed by accumulated data and results from clinical studies indicating prevalence rates of psychiatric comorbidity and specific psychological reactions and disease perceptions in tuberculous patients. Afterwards, we performed a review of the literature on psychiatric disorders in patients receiving antituberculosis drugs, possibly resulting in comorbid states as well as in treatment interruption which compromises treatment efficacy leading to cases of high-grade drug resistance.

Finally, we summarized factors affecting treatment adherence, and we concluded that management of psychiatric complications, especially depression which is more prevalent in tuberculosis, is necessary and possible without compromising antituberculosis treatment.

## 3. Results 

The literature review yielded in sum 135 articles: 4 on epidemiology, 3 on the past medical literature, 59 on results from clinical studies on psychiatric comorbidity in TB patients, 12 on psychological reactions and disease perceptions, 28 on psychiatric disorders in patients receiving anti-TB drugs, 17 on factors affecting medication nonadherence, and 12 on interactions involving TB medications and psychiatric medications. The overall citations are many more since we reviewed the reference section of these retrieved articles.

## 4. Psychiatry and Tuberculosis

### 4.1. A Review of the Literature

The psychological aspects of tuberculosis have always been a topic of interest over the centuries [180–182] and the association of tuberculosis and mental illness became a subject of statistical analysis as early as 1863 [183]. Clouston analyzed the deaths from tuberculosis in an Edinburgh asylum, after comparing figures from La Salpetriere and a New York state asylum and raised the question if conditions of living in asylums favor the development of tuberculosis or there is a special relationship between tuberculosis and insanity. Later, studies [184] in New York state hospitals indicated that prevalence rates of tuberculosis, diagnosed by X-rays findings, increased with the length of time between first admission and the X-ray survey, emphasizing the role of the contagious factor [185]. 

Concerning psychopathology of tuberculosis, Jacobson firstly observed that some tuberculous patients are euphoric and appear totally unaware of the gravity of their illness [186]. This clinical observation was later rejected by other studies [187, 188]. When attempting to describe the personality of tuberculous patients some writers implied that the number of neurotics among them was greater than the number encountered among the general population [187–189], but figures are lacking. When figures do come up, Day [190] believed that 30% of patients he observed in an English sanatorium were “ill of mind,” Breuer [191] reported that for 34% of his patients, tuberculosis was “psychologically determined,” and Forster and Shepard [192] found that 31% of tuberculous patients in Cragmor Sanatorium were suffering from an “abnormal mental state.” Bobrowitz [193] mentioned that from 20% to 50% of patients of the Otisville Sanatorium in New York leave against advice mainly for psychological reasons and Ashmore and Bell [194] found that among veterans tuberculous patients who left against advice in their first year of treatment, chronic alcoholism, psychopathy, and ignorance, all played a part. Also, tuberculous patients have been psychoanalyzed by Jelliffe and Evans [195] who concluded that they were “childish, selfish, self-centered, irritable, dissatisfied, and ungrateful.” Wittkower and Todd [196] described the various states of mind in these patients: “constructive resignation and indifference, depression and anxiety, defiance and ultra-cheerfulness, resentment and apathy.”

Scattered through the literature, there are numerous reports suggesting that the natural history of tuberculosis is modified under the influence of emotional factors, and Hartz [197] noted that “an individual may react to life situations with an anxiety state or other personal behavior in such a way as to interfere with healthy living, and these reactions may thereby become a most significant factor in the onset and course of clinical tuberculosis.”

## 5. Psychiatric Comorbidity in Pulmonary Tuberculosis

### 5.1. Theoretical Considerations

There have been times when the high incidence of tuberculosis in mental illness was interpreted to mean that perhaps tuberculosis may cause mental illness or that mental illness forms a strong predisposition to tuberculosis [198]. 

The literature suggests the mutual influence and relationship between physical and mental illness, and many studies report the nature and prevalence of comorbid physical illness with severe mental disorders [199]. Research in this direction is mainly concerned with studying physical morbidity among psychiatrically ill individuals [200], but studies to delineate psychiatric profile of physically ill persons [201, 202] have also received attention.

Medical illness and chronic disease create multiple burdens for patients, including the necessity to deal with pain, suffering, reduced quality of life, premature mortality, financial costs, and familial emotional trauma [110]. The risk factors for mental health problems are complex [203]. Presence of medical illness [204], stigma, and discrimination [92] are major determinants of mental disorders, especially mood and anxiety disorders. Usually, the more serious the somatic disease, the more probable will be, to be accompanied by mood and/or anxiety symptoms of variable severity [86]; conditions arising after the somatic disease is diagnosed. Failure to manage such mental health problems increases the patients' probability of suffering from complications, even lethal.

The lifetime prevalence of mood disorder in patients with chronic disease is from 8.9% to 12.9%, with a 6-month prevalence of 5.8% to 9.4% [205, 206]. According to findings from worldwide research, 20% of patients with somatic disease suffer from major depression [207, 208]. 

In patients with pulmonary disease in particular, functionality may be severely impaired due to chronic psychogenic and somatic pain [209], frequent hospital admissions, and dependency from medical and nursing personnel. The observed higher prevalence of depression and anxiety in patients with chronic pulmonary disease [210]—compared to other chronic diseases—may be explained within this context.

The reasons for the frequent cooccurrence of psychiatric disorders and medical illnesses could be that the first represents reactions to illnesses and treatment or that they are a direct physiological consequence of the illness or complications of treatments (INH-induced psychosis) [83, 134].

Also, psychiatric disorders may coincide with medical illnesses, without being etiologically related to them, but they complicate the diagnosis and management and can alter their course. For diagnostic purposes, it is often difficult to determine if the vegetative symptoms of depression or somatic symptoms of anxiety are evidences of the psychiatric disorder or symptoms of medical disease, or both. 

Another reason for the frequent comorbidity is that there are commonly shared risk factors [19] for the development of a variety of psychiatric and medical disorders (smoking [211], low socioeconomic status [93], etc.).

Tuberculosis is a classic example of a disease with both medical and social dimensions, characterized by its close relation to poor socioeconomic conditions [110]. Increased risk [1, 105] of acquiring active disease occurs with alcoholism, smoking, intravenous drug abuse, diabetes mellitus, HIV infection, and other factors. The above-mentioned risk factors are very prevalent in psychiatric populations and increase risk of progression from latent TB to active TB [104]. 

Patients suffering from pulmonary tuberculosis are reported to have psychiatric disorders like depression [212], anxiety, psychosis [83], and also many psychosocial problems [37, 213] like increased smoking [74], increased alcohol consumption [87], divorce, and isolation from the family [36]. However, it is important to draw the line between a psychosocial issue and a diagnosable mental disorder in order to address the effective interventions which necessitate different knowledge and skills [214].

Psychopathology may be a very important negative factor to treatment adherence [169] for patients with somatic disease, and nonadherence to treatment is a major setback for effective tuberculosis control in the community, possibly responsible for the appearance of drug-resistant TB which is caused by inconsistent or partial treatment of the disease [215] and probably responsible for nonadherence with latent TB therapy, which may increase risk of progression from latent TB to active TB. Additionally, psychiatric disorders may hinder adaptation to chronic disease conditions, and it is known that adaptation is a crucial survival factor in chronic diseases [216]. 

Temporal and causative associations determine possible distinct categories of psychiatric conditions in TB patients (presented in Table 1).

## 6. Results from Clinical Studies

Research has shown that people infected with TB are more likely to develop mental and psychological problems than people not infected with the disease [217, 218]. To be afflicted with pulmonary, tuberculosis is a unique and painful experience in the biopsychosocial history of an individual, and the emergent stress contributes to psychiatric morbidity [59]. 

Depression, posttraumatic stress disorder (PTSD), and acute stress disorder are the most common stress-related conditions of TB patients [36, 219]. Reactions to the stressful situation brought about by the illness negatively affecting an individual's ability to work, in conjunction with social and respiratory isolation [220], lowered self-esteem, fear of spreading the illness to others, helplessness brought out by incapacitation due to chronic illness, and social stigma attached to this illness, are all plausible causes that one can postulate for depression and anxiety. Dependence on alcohol and other drugs could be the response to anxiety and depression [59].

The psychiatric morbidity of patients is considered to be a psychogenic reaction of neurotically predisposed people to their special situation and awareness that they are suffering from a severe and dangerous illness [221].

Prevalence studies (Table 2) reporting mean depression and anxiety rates of 46%–72% establish the need to address mental disorders in TB care [214].

Conversely, individuals with chronic mental illnesses present a series of risk factors that predispose them to extensive medical comorbidities [102, 222]. Prevalence rates of tuberculosis among psychiatric patients are presented in Table 3, indicating that programs serving people with severe mental illness should regularly screen participants for TB infection and evaluate them for chemoprophylaxis in order to avoid the risk of developing active TB.

## 7. Psychological Reactions and Disease Perceptions

People believe that tuberculosis occurring in an individual is always an interruption in life, physically, psychologically, economically, and socially. Observations from the early days of the tuberculosis epidemic, before scientific studies, report that after the initial shock from the diagnosis, there is often a brief or prolonged period of denial, followed by resignation and depression, leading to distorted perceptions about the illness. Patients were described to exhibit strong emotions like fear, jealousy, vindictive behavior, anger, noncooperation, guilt, or a sense of shame. Rarely, suicides occurred, especially when the rest of the family tried to segregate the afflicted in a distant institution, or even stooped to a complete abandonment [223].

The scene changed dramatically in the fifties when tuberculosis became highly curable and preventable, assuming that physical, mental, economic, and social trauma had been rendered minimal, even that social stigma had nearly disappeared and needed no attention [109, 224]. 

Unfortunately, stigma is seen to play a huge role in the experience of illness by TB patients, and it is believed that most TB patients even after full recovery from the illness feel the disease can never be completely cured [225]. According to Lawn [226] “patient perception about TB is usually stained, and patients permanently hold negative feelings towards the disease.” Disease-related stigma occurs when individuals with an illness are deemed undeserving of assistance and support from other individuals in the society (Schulte [227]). Over time, certain illnesses, such as TB, have been associated with reduced social status, and these negative reactions may impede coping and recovery [228]. The psychological adjustments to illness by a patient are negatively influenced primarily by the negative perceptions that other people within the society might hold (Fife and Wright [162]). 

In other words, TB often has an impact on the physical, social and mental wellbeing of TB patients ( Rajeswari et al. [109]), and another element of this impact is the perception of others in the community about TB, which influences the self-perception of TB patients. The self-perception of a TB patient is influenced by the existing health-related beliefs in the community, the culture of the community, which a TB patient lives in, and the expected health behavior of a TB patient by the community.

Although patient's perceptions about TB remain largely unknown [72], yet the literature shows a lot of reactions of TB patients to the disclosure of their diagnosis and these reactions included feelings of loneliness, depression, suicidal thoughts, fear, apathy, shock, concern, surprise (in relation to the lack of symptoms), and acceptation [109, 115]. The possible reasons for these emotions may be the stigma discrimination and social isolation attached to the disease or “physical rehabilitation, illiteracy, lack of knowledge of TB, or fear of loss of income on account of long duration of treatment.” [109]

Studies reporting patients' psychological reactions and disease perceptions about TB are summarized in Table 4.

Evidence also suggests the correlation between susceptibility to tuberculosis and specific personality traits [229], and studies indicate that in a large number of tuberculosis cases emotional conflict appears to inhibit recovery, and major life changes [189] provoke relapses.

Emerging disciplines like psychoneuroimmunology and neuroendocrinology could pave the way to better understanding of the subject and explain how psychological distress may decrease proliferation of lymphocytes and natural killer cell function and provide clues to recovery from disease and prevent relapse [223]. 

## 8. Psychiatric Disorders in Patients Receiving Antituberculosis Drugs

Psychiatric complications have been associated with antituberculosis therapy since the 1950s [116, 117, 122]. The possible environmental and genetic factors of anti-TB medication-induced adverse reactions have always been the matter of concern [230]. It is well documented that the risk of adverse reactions increases with age, malnutrition, and history of hepatitis [231, 232], human immunodeficiency virus infection, and hepatitis C virus infection [233]. Genetic factors like isoniazid-metabolizing enzyme gene polymorphisms [234] were studied a lot, but these studies also showed inconsistent results. Until now, the comprehensive study for environmental, genetic, clinical, and administrative factors has not been reported. 

More specifically, adverse reactions concerning neuropsychiatric complications have been reported, mainly with isoniazid (INH) [134, 235, 236], which is a first line drug and with ethionamide (ETH) [237, 238] and cycloserine (CS) [239, 240] (both second-line drugs, reserved for patients with drug-resistant tuberculosis) [164, 241]. Psychiatric disorders in patients receiving TB medications are presented in Table 5.

## 9. Discussion

Nonadherence to therapy by patients has been cited as the principal obstacle in eliminating tuberculosis [242]. Studies indicate that up to half of all of patients with TB do not complete treatment [243], which contributes to prolonged infectiousness, drug resistance [244], relapse, and death [245]. WHO defines “treatment default” (nonadherence) as a treatment interrupted for two consecutive months, and it is well documented that 30% of all patients who are under self-administered treatment do not adhere to the therapy in the first two or three months. 

Different approaches for ensuring medication adherence have been adopted, since 1991, and include Directly Observed Therapy Short Course (DOTS) [246], medication monitors [247, 248], and legal action [249, 250], (the use of involuntary detention for persistently nonadherent patients as a last resort) resulting in cure rates of >80% and default rates of <10% [251]. A review of articles published from 1966 through 1996 on DOT programs for TB treatment found that treatment completion rates were greater than 90% when therapy was supervised [252, 253]. Having a health care worker present to directly observe patients taking each dose of anti-TB medication has been proposed as the best way to ensure adherence to treatment, thereby diminishing the risk of transmission, relapse or reactivation, and drug resistance [254].

However, even with this approach, patient nonadherence to DOT still occurs [255]. One problem cited is that it is difficult to anticipate who will comply with treatment [256]. Various factors such as age, gender [257], alcohol and drug dependence [166], absence of symptoms, adverse effects of drugs, absence of educational programs, quality of communication between patients and health workers [258–260], health culture [261], beliefs [262, 263], incentives and transportation time [257], and poor economy [264] have been shown to be associated with nonadherence. In sum, social, cultural, and demographic factors [265] (including educational level and treatment literacy [266]), psychiatric illness, including substance abuse [267] (alcohol and drug) in addition to those related to medication and also to the process of health care delivery and most certainly previous history of nonadherence [166] have all been cited as the most important barriers to TB treatment adherence. 

The ability to predict poor medication adherence at initiation of treatment and identify patients at greater risk of dropping out could help in dealing with the problem [268]. Recently, a 30-item TB medication adherence scale (TBMAS) with a positive predictive value of 65.5% and a sensitivity of 82.9% was developed and incorporated the latest research in TB specific medication adherence, where predictors for adherence such as patient behavior and patient-provider interaction in TB treatment have been explored. The resulting tool will help TB medical professionals identify not only TB patients with poor adherence but also potential reasons for nonadherence and help them to design and implement targeted interventions to improve adherence [178].

A study that focused on adherence to DOTS, carried out in India, verified the need to focus research on addressing the disease from the perspective of patients and health professionals, who are the essential elements in this process [269]. In the encounter between health professionals and patients, DOTS could be an opportunity for the manifestation of subjectivities and to help patients with tuberculosis to recover their capabilities for life during regular consultations. At the same time, it allows identifying vulnerabilities and needs that can be dealt with, during the process so as to overcome them [270], which points to the need for actions within a multidisciplinary team, according to the biopsychosocial model of health and illness, where adherence is conceived as a process, not of imposition, but rather of exchange and meeting, one that uses the understanding of the context of patients' lives as a trigger to meet social and health needs [271]. 

The presence of psychopathology has been found to be one of the causes of nonadherence with therapy in chest conditions [32, 33]. 

Not only psychiatric patients are at risk of getting TB infection, as they are often homeless or have unstable housing conditions and lack food and security, but they also frequently fail to comply with treatment for the same reasons [67].

Individuals who are dealing with issues of substance abuse, HIV infection, mental illness, intellectual disability, and are also often homeless/under-housed are at much higher risk of contracting latent TB infection and of developing active TB disease. This increased risk may be explained by the existence of a number of challenges that increase an individual's vulnerability to tuberculosis, such as inadequate access to food, shelter, and income; substandard and overcrowded shelter conditions; forced migration of shelter users; preexisting health conditions (e.g., hepatitis C, compromised immune system); structural and attitudinal barriers to effective health care; problems in the corrections system [272] (i.e., prison conditions); and immigration and refugee issues (e.g., lack of identification to access health care during first few months). These challenges also increase the probability that individuals living in these conditions will be unlikely to adhere with TB treatment.

In an earlier study [71] on a tuberculosis population, it was found that 30.2% of the population had diagnosable mental disorders and none of these were recognized by the clinic staff. Such lack of knowledge can contribute to negative, pessimistic or victim-blaming messages to TB patients [273, 274], which fuels patients' distrust and can lead to problematic treatment [161]. The importance of a nonjudgmental, nonblaming stance is often cited as foundational for psychotherapeutic care in TB [275] along with the adoption of more power sharing with TB patients. Specific strategies are also identified, including education to help recognize mental disorder, training in psychotherapeutic strategies [276], and communication skills building. 

TB is a chronic illness, and research into chronic illnesses has indicated that psychological factors, particularly depression, and the patients' perceptions about their illness predict poor adherence. In order to maximize the rate of adherence, health workers involved in the management of these patients should develop a higher index of suspicion for possible psychopathology and utilize the available consultation/liaison psychiatric services [169]. 

Treating psychological problems in patients with tuberculosis may substantially improve treatment adherence. According to studies, DOTS programmes are more likely to achieve better TB control outcomes if they include interventions aimed at improving diagnosis of alcohol and substance abuse and treating it concurrently with TB, [277, 278] and according to DOTS-Plus Guidelines, for MDR-TB patients, all healthcare workers treating drug-resistant TB should closely work with psychiatric services because there is a high baseline incidence of depression and anxiety in these patients, often connected with the chronicity and socioeconomic stress factors related to the disease [279].

According to all studies, irrespective of regional and population differences a common major factor implicating treatment adherence is the presence of psychopathology, especially depression, among tuberculous patients [280], and the high incidence of depression among these patients necessitates effective management [281] in order to improve treatment adherence and overall quality of life of these patients [282]. Results from these studies advocate a more holistic approach [91] to healthcare programs with the inclusion of mental health services in order to provide pretreatment psychiatric assessment and necessary intervention and eventually reduce default rate in tuberculosis control programs [34]. 

Awareness of adherence is, as a complex behavioral issue, influenced by many factors [283] and lack of a comprehensive and holistic understanding of barriers to and facilitators of, treatment adherence is currently a major obstacle to finding effective solutions [253, 284]. Knowledge about the degree that each of these factors correlates with psychopathology, and contributes to nonadherence is lacking, and prospective cohort studies addressing the cause-effect relationship between risk factors and psychopathology could clarify such issues. 

Also, studies focused on human dimension [28] and on subjective experiences of health care consumers [285] may provide information on patient experiences of TB treatment adherence which may serve as a tool to better promote treatment and effectuate more patient-centered interventions [286]. 

Finally, randomized control trials investigating the effects of pharmacological and psychological interventions modified to address not only depression but also issues around adherence to treatment [287] and illness perceptions [288] need to be carried out. 

Studies addressing factors affecting treatment adherence in pulmonary tuberculosis patients are presented in Table 7.

## 10. Treatment of Comorbid Tuberculosis and Depression

Mood disorders seem to be particularly common in TB patients compared with those with other medical diagnoses. 

Currently, selective serotonin reuptake inhibitors (SSRIs) are recommended as the first-line treatment for depression and tend to be favored over other pharmacologic treatments such as tricyclic antidepressants (TCAs) and monoamine oxidase inhibitors (MAOIs) because of their relatively benign side effect profiles. 

However, in patients comorbid for TB, concerns have been raised over the potential for drug interactions between various SSRIs and isoniazid, based on the ability of isoniazid to inhibit monoamine oxidase in plasma [289]. Generally, the combination of SSRIs or TCAs with a drug that inhibits monoamine oxidase is contraindicated because of the potential to induce serotonin syndrome [149]. No reports of serotonin syndrome induced by combining SSRIs and isoniazid are published, and, currently, there is insufficient clinical evidence to definitively establish the potential for an adverse interaction between isoniazid and antidepressants [151, 153].

At the molecular level, there is evidence that isoniazid and SSRIs are metabolized by similar mechanisms [159]. Hepatic cytochrome P450 enzymes are largely responsible for metabolism of isoniazid, citalopram, fluoxetine, fluvoxamine, paroxetine, and sertraline. While it has not been definitively established which isoenzymes are implicated in the metabolism of isoniazid, CYP2E1, CYP1A2, CYP2C9, CYP2C19, and CYP3A are inhibited to varying degrees by isoniazid [154], and inhibition of these enzymes slows the elimination of coadministered drugs. All SSRIs appear to be metabolized by cytochrome P450 enzymes; however, the pharmacokinetic interactions of each drug are variable, and available evidence indicates that some SSRIs might be a better choice than others for concurrent treatment.

Clinically, significant drug-drug interactions involving TB medications, especially isoniazid and rifampin, and various psychiatric medications are presented in Table 6.

## 11. Conclusion

Tuberculosis remains a leading infectious cause of mortality worldwide.

Studies report high rates of depression and anxiety among tuberculosis patients most likely related to social stigma, inadequate social support, and the physiologic impact of chronic disease. The paper integrates information about how these psychosocial factors complicate adherence to drug regimens and emphasizes the importance of attention to mental health needs to ensure positive treatment outcomes.

## Figures and Tables

**Table 1:** Categories of psychiatric conditions in TB patients.

(1)	Psychiatric conditions arising after TB are diagnosed (as reactions to the medical illness or a direct physiological consequence of the illness)—according to DSM-IV: (i) adjustment disorder, (ii) mood disorders (e.g., major depression), (iii) anxiety disorders (e.g., PTSD, acute stress disorder, and GAD), (iv) somatoform disorders (e.g., pain disorders and neurasthenia), (v) delirium and other cognitive disorders, (vi) personality change due to a general medical condition. Differential diagnosis in this category is necessary between a psychosocial issue and a diagnosable mental disorder.

(2)	Psychiatric complications associated with antituberculosis therapy.

(3)	Preexisting psychiatric disorders potentially increasing risk of TB and risk of progression from latent TB infection to active TB (e.g., substance related disorders, psychotic disorders, mood disorders, and psychological factors affecting medical condition).

(4)	Coexisting psychiatric disorders exacerbated by TB, without necessarily being etiologically related but complicate the diagnosis and management and can alter its course (e.g., specific phobia).

(5)	Comorbidity as a result of commonly shared risk factors for the development of a variety of psychiatric disorders and TB (e.g., substance related disorders and low socioeconomic status).

**Table 2:** Prevalence studies of psychiatric comorbidity in Tb patients.

First author/ references	Study design	Measurement instruments	Results	Rates in background population or in the control group used in studies and statistical significance	Comments
Moudgil (1972) [44]	40 TB inpatients of the Sanatorium, in Punjab. The mean scores of TB patients were compared with the mean scores of patients suffering from (a) cardiac illness where surgery was indicated (4); (b) chest disease where surgery was indicated (4); (c) vasectomy cases (10).	(1) Maudsley Personality Inventory (modified) (2) Cornell medical Index (Health Questionnaire).	TB patients and vasectomy cases (which is of course a normal population) had lower scores on MPI tuberculosis, and vasectomy cases had lower scores on CMI.	The neurotic scores on MPI of cardiac patients awaiting surgery were the highest, followed by chest diseases of patients awaiting surgery. The “physical distress” section of CMI reveals that a patient of cardiac illness awaiting surgery scores highest followed by patients of chest Diseases. In Maudsley Personality Inventory and in Cornell Medical Index (Health Questionnaire), *P* = 0.05 or less.	About 56% of the male patients had a habit of drinking. Although scores on the “neurotic dimension” of MPI of chest diseases patients and TB patients are similar, there is a significant difference in scores on the “physical distress” section of CML It may be concluded from this fact that the difference in physical distress will not have a corresponding difference in neuroticism level.

Kuha (1975) [45]	100 tuberculous patients to analyze the effect of social background factors on the psychiatric and psychological examination.	Psychiatric interview and psychological tests (MMPI, Rorschach, and Wartegg).	No correlation between the social group variable and those obtained in the psychiatric interview or the MMPI test could be demonstrated.		The purpose of the study was to analyze the effect of social background factors on the psychiatric and psychological examination. On the basis of the projective tests, subjects in the lower social classes were considered more disturbed.

Dubey (1975) [46]	50 TB patients admitted to the TB Ward of K.G.'s Medical College and Gandhi Memorial and Associated Hospitals.	Rorschach test, TAT	Lack of emotional control, insecurity, anxiety, and depressive features were observed in 60% of the cases.	31% psychiatric morbidity in patients admitted in medical wards [47] psychiatric morbidity of 20% in their study of two general medical wards [48].	No significant differences were found on Rorschach test. On Thematic Apperception Test, more females projected fear of death and fear of being cast out of the social sphere.

Purohit (1978) [49]	96 inpatient proved male cases of pulmonary tuberculosis, in Udaipur.	Self-rating depression scale of Zung	52 out of 96 patients showed evidence of depression (i.e., 54.17%).	In primary care, clinics/center have estimated a prevalence rate of depression: 21%–40.45% [50–53].	Excluded those patients who had previous history of any psychiatric illness before developing pulmonary tuberculosis and patients developing psychiatric illness other than depression. Depression was related to the duration and severity of the illness.

Yadav (1980) [54]	272 pulmonary tuberculosis patients with positive sputum, in Agra.	Psychiatric screening schedule developed on the basis of Wing's Screening PSE/diagnostic labels according to ICD.	29.4% of psychiatric comorbidity (19.4% with a diagnosis of depression and 6.6% with anxiety).	24.4% of 258 patients to be suffering from a purely psychiatric problem (anxiety neurosis 12.8% and depression 10.1%) and an additional 12% to have an associated psychiatric disorder bringing the overall morbidity to 36%. [55] 36% psychiatric morbidity in patients seen in general practice (Outpatient departments) [56, 57].	Patients aged below 50 years, with a positive sputum.

Tandon (1980) [58]	100 tubercular patients/control group: patients undergoing treatment for long-term fever of any etiology except tuberculosis from a clinic of Tuberculosis and Chest Diseases Hospital, Allahabad.	Hamilton rating scale for depression.	32% of tuberculosis patients demonstrated the presence of depression.	7% control cases *χ* ^2^ = 23.2, df = 2, *P* < 0.001.	The depression was directly related to the duration of illness.

Mathai (1981) [59]	70 inpatients with TB matched to 70 inpatients with nontuberculous, bronchiectasis, from the sanatorium for chest diseases, Trivandrum.	Clinical evaluation was performed, and diagnosis was arrived at as per ICD-9.	28.87% of psychiatric comorbidity (15.7% with a diagnosis of depressive neurosis, 7% with anxiety neurosis, and 3% with alcohol dependence).	7.14% of patients with nontuberculous bronchiectasis were found to be abnormal in psychiatric terms *χ* ^2^ = 10.96, df = l, *P* < 0.001.	All patients had been on medication and followup for at least 6 months without any untoward reactions to ensure that the symptoms produced were not due to antituberculous medication per se/rule out CNS involvement.

Gupta (1981) [60]	60 patients of pulmonary tuberculosis/ and a matched control group of normal nonpatient healthy relatives were selected from TB Hospital, Bikaner.	Assessed by Present State Examination.	41.6% of patients of pulmonary tuberculosis have one diagnosable psychiatric disorder.	For male: *t* = 4.84, *P* < 0.001 For female: *t* = 6.88, *P* < 0.001.	41.6 % of patients with pulmonary tuberculosis have reported more than one-life event in preceding one year.

Natani (1985) [41]	150 patients of pulmonary tuberculosis admitted in Hospital for Chest Diseases and Tuberculosis, Jaipur.	Beck Depressive Inventory.	A depression rate of 49% in hospitalized tuberculous patients, which decreased with favorable response to chemotherapy but increased in those with persistently positive sputum, up to 64%.	31% of medical inpatients had psychiatric comorbidity (16% depression, 9% anxiety neurosis, and 5% organic brain syndrome) [47].	The depression was directly related to the duration of illness, severity of disease, and response to chemotherapy.

Meghnani (1988) [61]	110 hospitalised TB patients in a Chest Hospital in Jodhpur.	Hamilton rating scale for depression.	A depression rate of 53.6%.	41.9% of medical in patients had depression [62].	The depression was related to the duration of illness, and severity of the disease/excluded those patients who had previous history of any psychiatric illness before developing pulmonary tuberculosis and those with severe illness and on specific anti-TB meds.

Immerman (1988) [63]	232 patients with new cases of tuberculosis.		Neurotic disturbances have been diagnosed in 64.7% of the patients, with asthenic and depressive syndromes constituting 84.7% of all mental disorders.		Premorbid personality peculiarities are significantly correlated with the incidence and nature of mental disorders being most frequent in individuals with asthenic and psychasthenic features. Specific antituberculosis therapy fails to control neurotic disturbances by the end of the main course of the inpatient treatment in 51.5% of the patients which poses a question about the necessity of psychotropic therapy.

Singh (1989) [64]	100 cases of chest diseases: 50 tubercular and 50 nontubercular/ control group, admitted to a Chest Hospital, in Kanpur.	Cornell Medical Index Questionnaire.	70% in the study group were found to have psychiatric problems/ depression was prevalent in the study group (77%).	56% in the control group were found to have psychiatric problems/anxiety state was most common in the control group (57%).	The prevalence of psychiatric illness was higher in females than males, high between 15 and 44 years (91%) and more in the low socioeconomic group, illiterates, and semiliterates; more in housewives, unskilled workers, and large and joint families. Higher psychiatric morbidity was observed in chronic, far-advanced and resistant tuberculosis patients.

Vinogradov (1991) [65]	To examine the mental status and personality traits of 61 patients with newly diagnosed pulmonary tuberculosis of limited extent.	MMPI	Revealed the following general types of a response to the disease: alienation from the people around, depression reaction (18%), negative attitude to treatment (16.1% of the patients refused treatment and 13.1% refused surgical treatment), social adaptation impairment, neglect of the generally accepted behavior patterns, and schizoid personality traits. Along with this, the individual forms of a response to disease detection were determined. They were manifested by a number of symptom complexes: hypochondriac (13.6%), anxiety depression (18.4%), and paranoid (9.1%).	The mental status and the types of response were shown to differ from the same reactions in somatic patients with other abnormalities.	These mental disorders gravely affected the patients and made treatment of the basic disease more complicated. A long-term conservative treatment aggravated depression, hysterical, and schizoid personality traits.

Westaway (1992) [21]	100 black hospitalized tuberculosis (TB) patients in Pretoria, South Africa.	The 13-item shortened BDI and the Rosenberg Self-Esteem scale.	A depression rate of 68%	The prevalence of psychiatric disorder in primary care was 21.3%. Depressive neurosis (51.7%) and anxiety neurosis (36.3%) were the most common disorders [66].	Self-esteem scores dropped in accordance with category of depression.

Fullilove (1993) [67]	121 TB patients seen in a Medical Center in New York.		22% psychiatric comorbidity.	Prevalence of major psychiatric disorders in primary health care is 11.9% in US (1995). 6.3% of patients attending primary care facilities suffer from depression [68] in acutely ill medical inpatients 27.2% prevalence of psychiatric disorders; major depressive disorder was present in 5.1% [69].	

Chaudhri (1993) [70]	153 cases of pulmonary tuberculosis who had been defaulting in their treatment patients and 91 freshly diagnosed (to serve as controls).	Cornell Medical Index (CMI) to monitor psychiatric illness and Eysenck's Personality Inventory (EPI) for personality evaluation.	Depression followed by anxiety neurosis was the commonest of the psychiatric disorders. In EPI, the defaulters had more of neurotic personality compared with controls, while the extrovert traits were about equal.	Significantly higher proportion of defaulters had abnormal CMI scores.	The study suggested that identification of the patients at the start of treatment could help in reducing default because depression and anxiety neurosis could be treated along with tuberculosis.

Aghanwa (1998) [71]	53 outpatients with pulmonary tuberculosis seen in a Nigerian chest clinic compared to 20 long-stay orthopedic patients with lower limb fractures and 20 apparently healthy controls.	30-item General Health Questionnaire (GHQ-30), the Present State Examination (PSE), and a clinical evaluation based on ICD-10.	30.2% prevalence of psychiatric disorders/11.3% prevalence of depression.	15% prevalence of psychiatric disorders in the orthopedic group and 5% in healthy controls.	The types of psychiatric disorders encountered included mild depressive episode, generalized anxiety disorder, and adjustment disorder (ICD-10). Psychiatric morbidity was higher in tuberculosis patients with low educational attainment.

Bhatia (2000) [72]	50 outpatients attending a TB Hospital in Delhi.	EPQ-R neuroticism scale/Dysfunctional Analysis Questionnaire (DAQ).	On neuroticism scale 78% of patients scored significantly.		The degree of neuroticism correlated significantly with scores on subscales of DAQ. Higher neuroticism showed higher psychosocial dysfunctioning.

Aydin (2001) [73]	157 male inpatients: 42 with recently diagnosed (RDtb), 39 with defaulted (Dtb), 39 with multidrug resistant tuberculosis (MDRtb), and 38 with COPD, in Ankara, Turkey.	Composite International Diagnostic Interview (CIDI)/Brief Disability Questionnaire.	Depression and/or anxiety comorbidity was 19% for RDtb, 21.6% for Dtb, and 25.6% for MDRtb.	Depression and/or anxiety comorbidity was 47.3% for COPD.	Patients with psychiatric comorbidity had higher disability scores than the groups without psychiatric comorbidity.

Manoharam(2001) [74]	52 TB patients attending a primary care centre in Vellore.	Revised Clinical Review Schedule for assessing psychiatric morbidity and the Short Explanatory Model Interview to identify patients' perspectives of their illness.	17.3% of subjects satisfied the International Classification of Diseases 10 Primary Care Criteria for psychiatric disorders. Depression was the commonest disorder (13.5%).	Studies done in primary care clinics/center have estimated a prevalence rate of depression of 21%–40.45% [50–53]. 36% psychiatric morbidity in patients seen in general practice (Outpatient departments) [56, 57]. Prevalence of major psychiatric disorders in primary health care 22.4%, depression (9.1%) [68].	1/4 of patients defaulted during 5 months treatment while just a third completed 6 months course of therapy.

Bhasin (2001) [75]	103 tuberculosis eases and a similar number of age, sex matched controls to find out the difference in illness behavior profile of the two groups.	Illness Behavior Questionnaire (IBQ).	TB patients exhibited features pertaining to general hypochondriasis, affective inhibition, and affective disturbance more than controls.	Denial of problem was seen more in controls. The differences between the two groups were statistically significant.	The tuberculosis patients were receiving treatment from two DOTS centres in East Delhi, and the controls were from the same locality. A valid illness behavioral profile of these patients to be used as an adjuvant to the implementation of the revised tuberculosis control programme.

Furin (2001) [76]	A retrospective record review of 60 patients who had received individualized therapy for MDR-TB.	Defined using DSM-IV criteria.	Depression was the most frequent baseline finding, occurring in 38.3% of the patient population and alcoholism in 3.3%.		Side effects of medication include: depression newly diagnosed in 18.3% patients after a median of 8.5 months, anxiety in 11.7%, and psychotic symptoms in 10%.

Rogacheva (2002) [77]	206 patients with pulmonary tuberculosis and mental disorders from the Kirov Region compared with 154 control patients with pulmonary tuberculosis without mental disorders.		In both group, males fell ill with tuberculosis in the prime of their life, whereas females did at their old age. Males with mental disorders are more susceptible to tuberculosis than mentally healthy patients.		In contrast, females with mental disorders are much less susceptible to tuberculosis than mentally healthy patients. Women of reproductive age are the least prone to tuberculosis particularly in the presence of mental disorders.

Lukashova (2002) [78]	110 adolescent patients with respiratory tuberculosis and 89 healthy adolescents aged from 13 to 17 years.		The adolescent patients differed from healthy individuals by inadequate communicability, sensitive, liability to accumulation of negative emotions, by bad need for support and feelings to be taken hard, by marked internal strain, and yearning for showing his/her individuality.		This also had led to the lower behavioral range, made social adaptation difficult, promoted the susceptibility to stress exposure, and increased a risk for a disease.

Yang (2003) [79]	132 patients with tuberculosis and 71 healthy volunteers.	Symptom Checklist 90 (SCL-90) and Social Support Rating Scale (SSRS).	Somatization, obsessive compulsiveness, anxiety, phobic anxiety, and paranoid ideation, psychoticism and the mean of positive factors of SCL-90 of the tuberculosis group→/. The SSRS results of subjective and objective supports and total score of social supports of the tuberculosis group*	→were significantly higher than those of the control group/. *were much lower than those of the control group.	

Sukhova (2003) [80]	253 patients with fibrocavernous pulmonary tuberculosis and 178 patients with infiltrative pulmonary tuberculosis.	Standard multifactorial personality study and the Lusher tests, special questionnaire surveys.	Irrespective of the duration of the disease, specific psychological peculiarities, and altered behavior and attitude to themselves and others appear in both males and females, leading to the socially dangerous manifestation of behavioral aggression.		The study has developed a procedure to prevent the manifestation of aggressive behavior in patients with pulmonary tuberculosis. Goal-oriented correction prevents distresses resulting in decompensation.

Sukhov (2003) [81]	152 males with fibrouscavernous pulmonary tuberculosis and 123 males with infiltrative pulmonary tuberculosis.	Multifactorial psychological personality testing.	The psychological characteristics were more impaired in male patients with chronic pulmonary tuberculosis. Life quality in male patients with chronic pulmonary tuberculosis is still worse than in those with first diagnosed pulmonary tuberculosis.		By recognizing that life quality is an integrative indicator of the functional parameters of health and the social and psychological parameters of living standards and life way: life quality in all male patients with pulmonary tuberculosis may be considered to be low.

Vega (2010) [40]	A retrospective case series was performed among the first 75 patients to receive individualized MDR-TB therapy in Lima, Peru.	Based on DSM-IV criteria.	Baseline depression and baseline anxiety were observed in, respectively, 52.2% and 8.7% of this cohort. The incidence of depression, anxiety, and psychosis during MDR-TB treatment was 13.3%, 12.0%, and 12.0%, respectively.	A 6.7% prevalence rate of depression in the general population of Lima, Peru [82].	Baseline rates of anxiety and psychosis were comparable to those of the general population of Lima.

Vhandrashekar (2012) [83]	100 patients hospitalized for pulmonary tuberculosis in Bangalore.	MINI-International Neuro Psychiatric Interview Scale.	46% of psychiatric morbidity, majority is depressive disorders (36%) followed by anxiety disorders (24%)/comorbidity of depressive and anxiety disorders in 16% of patients.	31% psychiatric morbidity in patients admitted in medical wards [47] psychiatric morbidity of 20% in their study of two general medical wards [48].	Depressive disorders are more in lower socioeconomic groups, patients with longer duration of tuberculosis illness, who stayed in hospital for longer duration and patients receiving non-RNTCP drugs. Anxiety disorders are more in lower educated group, tuberculosis associated with complications and patients with longer hospital stay.

Aniebue (2006) [84]	105 patients affected by tuberculosis seen at the chest clinic of University of Nigeria Teaching Hospital.	Zung Self-rating depression scale.	41.9% of patients had depressive symptoms.	The prevalence of psychiatric disorder in primary care was 21.3%. Depressive neurosis (51.7%) and anxiety neurosis (36.3%) were the most common disorders [66].	Being widowed or single, increasing age, unemployment, duration of illness, duration of treatment, and being accompanied to hospital increased the prevalence of depressive symptoms amongst TB patients. However, unemployment, being accompanied to hospital, and duration of treatment significantly increased prevalence of depression in affected patients.

Eram (2006) [39]	100 patients attending tuberculosis clinic under Rural and Urban Health Training Centre in Aligarh.	Revised Clinical Review Schedule for assessing psychiatric morbidity and the Short Explanatory Model Interview to identify patients' perspectives of their illness.	30% had anxiety or tension while 26% had loss of interest for life or depression. 6% of patient denied the diagnosis while 20% of them could not explain how they felt.	Prevalence of major psychiatric disorders in primary health care: 22.4%, depression (9.1%) [68].	The negative reaction like tension and depression were more common in less educated patients. Similarly, this negative reaction was also more prevalent in low socioeconomic class compare to educated and higher socioeconomic class.

Gelmanova (2007) [85]	A retrospective cohort study with 207 participants enrolled in the DOTS treatment programme was included in the analysis of MDR acquisition.		8.8% of the patients in the cohort defaulted on therapy and 15.6% took fewer than 80% of their observed prescribed doses. 6.3% acquired MDR during the study period.		Substance abuse was identified as the only factor that was strongly associated with nonadherence with odds ratios for baseline alcohol dependence—4.38 (95% CI: 1.58–12.60); reported alcohol use in a patient during therapy—6.35 (95% CI: 2.27–17.75); and intravenous drug use—16.64 (95% CI: 3.24–85.56). The adjusted odds ratio of nonadherence for those with any kind of substance abuse was 7.30 (95% CI: 2.89–18.46). Substance abuse was also strongly associated with default, with an odds ratio of 15.57 (95% CI: 3.46–70.07) among those with baseline alcoholism and 5.14 (95% CI: 0.87–30.25) for those with reported alcohol use. Patients with any form of substance abuse had an adjusted odds ratio for default of 11.20 (95% CI: 2.55–49.17).

Moussas (2008) [86]	132 patients with pulmonary disease (42 were diagnosed with BA, 60 with COPD, and 30 with TB).	Beck Depression Inventory (BDI), Spielberger's state-trait anxiety scale.	In TB patients, mean anxiety was 40.67, SD = 9.19, and mean depression was 9.93, SD = 7.71.	In COPD, mean anxiety was 45.87 and mean depression was 15.48. In BA, mean anxiety was 43.67 and mean depression was 14.31. 28.1% of patients hospitalized in general medical or surgical hospital wards had depression [74, 87].	Patients with COPD had the higher depression scores, followed by patients with BA, whereas patients with TB had the lowest depression scores. Anxiety was higher in patients with COPD compared to patients with TB.

Husain (2008) [88]	108 consecutive outpatients with tuberculosis attending the TB clinic at the chest disease department in a Medical Centre in Karachi, Pakistan.	Hospital Anxiety and Depression scale (HADS) and the Illness Perception Questionnaire (IPQ).	46.3% were depressed, and 47.2% had anxiety.	Mean prevalence of anxiety and depression in Pakistan found to be around 34% (range 29–66% for women and 10–33% for men) in community based population.	Depression and lack of perceived control over illness in those suffering from tuberculosis are reported to be independent predictors of poor adherence.

Ntarangwi (2008) [89]	A cross-sectional consecutive study with 160 TB patients attending a Chest Disease Hospital in Nairobi, Kenya.	Beck Depression Inventory (BDI), socio-demographic Questionnaire (SDQ).	61% of respondents had clinically significant depression presented as follows, 22.6% had mild depression, 25.2% had moderate depression, and 13.2% had severe depression.	The prevalence of psychiatric disorder in primary care was 21.3%. Depressive neurosis (51.7%) and anxiety neurosis (36.3%) were the most common disorders [66].	

Issa (2009) [90]	65 patients with TB attending the DOTS outpatient clinic in a university teaching hospital in Nigeria.	Nine-item Patient Health Questionnaire (PHQ-9).	27.7% of patients had depression.	The prevalence of psychiatric disorder in primary care was 21.3%. Depressive neurosis (51.7%) and anxiety neurosis (36.3%) were the most common disorders [66].	

Bansal (2010) [34]	214 outpatients registered at DOTS centre in Kanpur, India.	Cornell Medical Index and 16PF-Test FORM-A.	82.2% had psychiatric comorbidity; 85.2% had anxiety neurosis, and 14.8% had depression. On personality assessment, 54.1% were anxious, 26% introverts, 15.8% extroverts, and 4.1% had other personality traits.	Prevalence of major psychiatric disorders in primary health care 22.4%, depression (9.1%) [68].	Patients with neurotic trait defaulted more as compared to other personality traits. On multivariate analysis, smoking habit and alcoholism were strongly associated with default whereas age, sex, socioeconomic class, and literacy were not.

Aamir (2010) [82]	65 newly diagnosed Pulmonary TB outpatients at the District TB Control Office and TB Centre in Haripur.	Hospital Anxiety and Depression Scale (HADS).	72% of TB patients had severe/moderate level of anxiety and depression.	Prevalence of major psychiatric disorders in primary health care 22.4%, depression (9.1%) [68].	22% of TB patients with comorbid anxiety and depression showed multidrug resistance (MDR-TB). 50,7% adhered to the treatment after consulting a psychiatrist.

Kruijshaar (2010) [91]	61 patients at three clinics in London, at diagnosis, and 2 months into therapy.	Generic health related quality of life (Short Form 36 [SF-36] and EQ-5D) and psychological burden (State-Trait Anxiety Short-Form, Center for Epidemiologic Studies Depression Scale, worry items).	Respondents' mean anxiety and depression scores were high at diagnosis (84.2% and 38.6%, resp.), and anxiety scores remained high at followup.	24.8% prevalence of psychiatric disorders and depressive disorder was present in 16.9%, in primary care in London [69]. At diagnosis, scores for all eight SF-36 dimensions were significantly worse than UK general population norm.	Although treatment significantly improved patients' health status within 2 months, scores for many domains remain below UK norm scores.

Deribew (2010) [92]	A cross-sectional study in three hospitals in Oromiya regional state of Ethiopia with 155 TB/HIV coinfected and 465 noncoinfected HIV outpatients.	Kessler 10 scale.	Common mental disorders (CMD) was present in 63.7% of the TB/HIV coinfected patients and in 46.7% of the noncoinfected patients.	Common mental disorders account for 9.8% of the global burden of diseases in low and middle income countries (LAMIC). 1/3 of all patients seen in primary care facilities in LAMIC present with CMDs [93].	Individuals who had no source of income, day laborers and patients who perceived stigma and rate their general health as “poor” were more likely to have CMDs.

Naidoo (2010) [38]	166 with TB (36.7% were also HIV positive) who were attending a public health clinic in the Cape Metropole area of South Africa.	BDI, Social Network Support Questionnaire, a semistructured questionnaire designed to assess helplessness.	64.3% of patients had depression (mild mood disturbance—26.1%, borderline clinical disturbance—10.3%, moderate depression—15.8%, severe depression—9.7%, and extreme depression—3.6%).		10.9% of the group in the study had feelings of helplessness and inadequate social support.

Sulehri (2010) [94]	A cross-sectional study with 60 TB patients admitted in the Department of Chest Medicine TB Hospital in Faisalabad, Pakistan.	Beck depressive inventory.	Depression was present in 80% of TB patients (86% in males and 71% in females).	Mean prevalence of anxiety and depression in Pakistan found to be around 34% (range 29.66% for women and 10.33% for men) in community based population.	Main causes of depression among the male TB patients were altered social relationship and among female patients TB stigma. Depression had adverse effect on drug compliance and TB treatment.

Panchal (2011) [42]	600 patients of pulmonary TB admitted in Hospital for Chest Diseases and Tuberculosis, Jaipur.	Beck depressive inventory.	Depression was present in 82% in female tuberculous inpatients and in 52.6% in males immediately after the diagnosis.		The depression was related to the duration, severity of illness, and response to chemotherapy, meaning that rate of depression decreased to 72.5% in those who responded favorably to chemotherapy, but in failures of treatment depression further increases and rises to 86%.

Adina (2011) [95]	60 patients treated for tuberculosis in Pulmonary Hospital or Sanatorium Savadisla, Romania.	Beck Depression Inventory—BDI, State Trait Anxiety Inventory STAI, and Illness Perception Questionnaire—IPQ.	6.78% for severe depression, 32.2% for moderate depression, and 32.2% for severe and 40.68% for moderate anxiety.		For patients at first admission in hospital (new case), the anxiety score is less than for chronic patients or with multiple admissions. Depression was positively correlated with anxiety.

Prakash (2011) [96]	50 TB outpatients in followup or new from a Hospital in Patna.	MINI international neuropsychiatric interview.	Common mental disorders in 76% of patients (39.47% depression, 42.1% GAD, and 13.15% organic brain syndrome/52.63% with suicidal ideation)	24%–36% rates of depression in patients admitted in medical wards for general medical conditions Moffic HS, Paykel ES (1975) and Cavanaugh (1983) [96].	Excluded were patients with previous psychiatric or drug history.

Mayowa (2011) [97]	88 TB outpatients and 81 family members visiting the DOTS Centre at University College Hospital Ibadan Centre in Nigeria.	Hamilton Depression Scale.	The prevalence of depression was 45.5% among patients.	Prevalence of depression was 13.4% among family members.	Depression was more prevalent among patients that were elderly (*P* = 0.001), with extensive disease (*P* = 0.01), of long duration (*P* = 0.03), those with category 2 tuberculosis (*P* = 0.003), those from a nuclear family (*P* = 0.01), and patients that were unmarried (*P* = 0.02).

Tangyu Xiu Lu (2011) [98]	426 cases of TB outpatients.	A psychological assessment questionnaire.	66.2% of patients presented with psychological problems.		

Williams (2012) [99]	A descriptive Study with 500 pulmonary tuberculosis patients undergoing DOTS therapy in selected areas of district Jalandhar, Punjab.	Structured checklist to measure psychological and sociological problems of pulmonary TB undergoing DOTS therapy. It consists of 20 items to which respondents were expected to answer yes/no (any other specific answer).	Among psychological problems pulmonary tuberculosis patients undergoing DOTS therapy showed maximum results in category of sadness due to disease (76.2%), followed by feeling emotionally disturbed (73.2%), followed by patients loosing temper while dealing with others (53.2%), and in the presence of sleep disturbance (51.2%).		Among sociological problems, patients with pulmonary tuberculosis undergoing DOTS therapy showed maximum results in category of finding difficulty to continue job (41.2%), followed by preferring stay alone (39.6%), followed by not finding cooperation from colleagues at work place (25.2%), followed by feeling of isolation by friends and relatives (24.8%), and loss of job due to disease (23.6%).

Peltzer [100]	A cross-sectional survey of 4900 tuberculosis public primary care patients within one month of initiation of antituberculosis treatment.	Kessler-10 item scale 10-item Alcohol Disorder Identification Test (AUDIT).	Overall prevalence of psychological distress in this study was 32.9% (K-10 ≥ 28) and 81.1% (K-10 ≥ 16), respectively. The authors in this study recommend the use of a cutoff score of 16 for use in South Africa, particularly, within the public sector health clinics in order for cost-efficient treatment programmes to be implemented on a large scale. 23.3% were hazardous or harmful alcohol users 31.8% of men and 13.0% of women were found to be hazardous drinkers, and 9.3% of men and 3.4% of women meet criteria for probable alcohol dependence (harmful drinking) as defined by the AUDIT.	The prevalence of psychological distress in this study is inline with the prevalence rates of depression or common mental disorders in most other studies with tuberculosis patients. 46%−80% in LMICs rates of hazardous or harmful alcohol use. In general, public primary care patients in South Africa 13.3% and 19.2% and in a national population-based survey in South Africa (9%) [93].	46.3% perceived their health status as fair or poor. Adherence to TB medication, 33.9% indicated that they had missed at least 10% their medication in the last 3-4 weeks. In this study, there was no association found between TB and HIV treatment nonadherence and common mental disorders as found in other studies. Alcohol use disorders in tuberculosis patients in low and middle income countries: Russia: 24–62% alcohol abuse/dependent, India: 14.9–32% alcohol abusers/alcoholics, Brazil: 14–24% alcohol abusers, South Africa: 31–62% alcohol misuse.

Peltzer (2012) [101]	4900 public primary care adult patients (either new or retreatment cases) from clinics in high TB burden districts from three provinces in South Africa.	Brief screening self-report tools were used to measure PTSD symptoms, psychological distress (anxiety and depression) and alcohol misuse.	The prevalence of PTSD symptoms was 29.6%.		Factors that predicted PTSD symptoms were poverty, residing in an urban area, psychological distress, suicide attempt, alcohol and/or drug use before sex, unprotected sex, TB–HIV coinfected, and the number of other chronic conditions.

**Table 3:** Prevalence studies of TB comorbidity in patients with chronic mental disorders.

First author/references	Study design	Results	Comments
Collins (1956) [102]	700 psychiatric inpatients in a mental hospital.	25 (14 with the diagnosis of schizophrenia) of them suffered from pulmonary tuberculosis and were under surveillance in the sanatorium.	

Ohta, 1988 [103]	3,251 patients residing in Nagasaki city and diagnosed as schizophrenia between 1960 and 1978.	Eighty-two of the patients had tuberculosis.	The incidence rate of tuberculosis was significantly higher than that of the general population for both male and female schizophrenic patients.

Lopez (1994) [19]	43 psychiatric patients at a general hospital at time of discharge.	19% were PPD positive at time of discharge, with 2 patients requiring a course of isoniazid prophylaxis.	

Saez (1996) [12]	85 men discharged to community living by the on-site mental health program between 1990 and 1992, in NY.	36.7% were PPD positive, 11.1% had inconclusive results, and 6.7% had active TB.	All HIV-positive men, PPD-positive or inconclusive and all had active TB.

McQuistion (1997) [20]	71 participants in a psychiatric day program of New York City teaching Hospital were given a skin tuberculin test.	17% had positive results. Of the 20 patients with mood disorders, 6 patients (30%) had positive PPD results, accounting for half of all positive findings in the total group. Only 6 (14%) of 41 patients with psychotic disorders demonstrated positive PPD results.	11 of the 12 infected subjects had experienced at least one of seven risk factors of tuberculosis infection (immigration, intravenous drug use, alcohol abuse, history of homelessness, HIV seropositive, known exposure to a person with active TB disease, and currently living in a congregate care setting).

Sanchez (1998) [104]	Using a screening questionnaire to assess the likelihood of TB infection in a random sample of 187 patients seen by a psychiatric emergency service, based on exposure to risk factors (emigration, age over 32 years, male gender, prior psychiatric hospitalizations, injection drug use, alcohol abuse, known exposure to a person with active tubercular disease, and concurrent illness).	83% were older than 32 years, 61% were male, 48% were immigrants, 44% had prior psychiatric hospitalizations, 35% abused alcohol, 35% were homeless, and 17% admitted to intravenous drug use.	While these figures did not reach statistical significance, there appeared to be a trend between various risk factors and TB infection.

Sanchez-Mora (2007) [105]	154 psychiatric patients at a long-term mental institution.	4.5% prevalence rate of tuberculosis.	

Pirl (2005) [106]	535 patients admitted to a state psychiatric Hospital, in Boston.	20.2% rates of positive tuberculin compared with 5% screened positive in the US general population, *z* = 16,12 *P* < 0.001.	Independent risk factors for markers of disease included age, immigrant status, homelessness, and history of substance use. The study confirms the alarmingly high occurrence of positive tuberculin skin tests.

Hashemi (2009) [107]	215 psychiatric patients in a long-term mental institution, in Hamedan, Iran.	28.8% rate of positive PPD results, 40.3% of them had radiographic evidences of inactive pulmonary tuberculosis.	In Iran, higher prevalence rates were found in prisoners, drug abusers, and hospital employees (50%, 66.7%, and 36.2%, resp.); however, they imply lower prevalence rates (1.6–14.2%) in low-risk groups.

Cavanaugh (2012) [108]	75 residents exposed to TB at the assisted living facility for adults with mental illness, in Florida.	88% were infected. By comparison, the prevalence of latent TB infection among reported contacts of pulmonary TB patients with positive sputum smears in the United States has been estimated at 20%–30%.	An elevated risk for TB infection among adults with mental illness and a risk for sustained transmission when they inhabit crowded congregate settings.

**Table 4:** Studies reporting patients' psychological reactions and disease perceptions about TB.

First author/ references	Reports from studies
Eram [39]	Tuberculosis is a disease both of individual and society and patients' first reactions to the diagnosis were tension/anxiety (30%), loss of interest/depression (26%), denial (6%), could not explain how they felt (20%), and only 18% were hopeful of cure.

Rajeswari [109]	The initial reaction of patients to the disclosure of the diagnosis was worry (50%) and suicidal thoughts (9%).

Deribew [92]	People with perceived stigma may have a low self-image and be socially isolated which may predispose them to common mental disorders.

Jaggarajamma [110]	Perceived stigma which refers to the fear of discrimination or acceptability was higher than enacted stigma, which refers to actual discrimination or acceptability.

Courtwright [111]	The most common cause of TB stigma is the risk of transmission from TB infected individuals to susceptible community members but also because of its association with HIV, poverty, low social class, malnutrition, or disreputable behavior. Also, TB stigma had a more significant impact on women and on poor or less-educated community members, which is especially concerning given that these groups are often at higher risk for health disparities [112]. TB stigma may, therefore, worsen preexisting gender- and class-based health disparities, and it is perceived to increase TB diagnostic delay and treatment nonadherence [37, 113].

Naidoo [38]	People who have a lifelong or infectious disease and who have limited social, psychological, and economic resources find it extremely difficult to maintain a reasonable quality of life and that helplessness [114] and depression have a negative influence on adherence to treatment.

Marra [115]	There are numerous aspects of active TB that may lead to a reduction in the quality of life, such as prolonged therapy with multiple, potentially toxic drugs that can lead to adverse reactions in a significant number of patients. Also, there is considerable social stigma associated with active TB leaving the individual feeling shunned and isolated from their friends and families, and there is a lack of knowledge regarding the disease process and its treatment which may contribute to feelings of helplessness and anxiety.

**Table 5:** Psychiatric disorders in patients receiving TB medications.

Anti-TB drug	Reported adverse event	Frequency of event	References
Isoniazid (INH) or iproniazid (IPH)	Toxic psychoses developed while under treatment with isoniazid or iproniazid in combination with other antituberculous drugs	5 cases seen at Charity Hospital of Louisiana, at New Orleans	[116]

Cycloserine	Showed some type of neurologic or psychiatric disturbance of varying severity	15 out of 30 TB patients 6 showed severe disturbance of function, either manifested by mounting signs of central nervous system instability (grand mal convulsions) or in borderline or outright psychosis.	[117]

Isoniazid and ethambutol	Peripheral neuropathy is associated with the use of isoniazid	In approximately 17% of patients using doses of 300 mg daily and lower frequency to the use of ethambutol	[118]

Isoniazid and ethambutol	The optic neuritis manifests with reduced visual field or acuity or color vision	Uncommon during the use of isoniazid and ethambutol/is related to generally at high doses or prolonged use	[118]

Isoniazid	The behavioral disorders, changes in the rhythm of sleep, reduced memory, and psychosis have been described for the use of isoniazid. Seizures and coma are described by the excessive intake of isoniazid.	Alcoholism, diabetes mellitus, malnutrition, and uremia are all predisposing factors for neurological and psychiatric disorders listed here.	[118]

streptomycin	The toxicity acoustic (or vestibular) is a complication related to the use of streptomycin.		[118]

Isoniazid	Minor adverse effects. Changes in behavior: headache, insomnia, euphoria, agitation, anxiety, and somnolence can occur in patients receiving isoniazid		[119]

Isoniazid	Major adverse effects: Psychosis, convulsive seizures, mental confusion, and coma. Attempted suicides have been reported to occur among patients using isoniazid	In patients receiving isoniazid, neurological and psychiatric manifestations are less common, more severe, and often difficult to diagnose. The differential diagnosis with tuberculous meningitis and hepatic encephalopathy should be established.	[119]

Isoniazid	Out of the five psychotics, three were manic, and two were depressive. Among the six neurotics, three were depressive and one each of anxiety, obsessive compulsive, and phobic neurosis	11 (five psychotics and six neurotics) out of 732 in patients of the hospital for tuberculosis and chest diseases, symptoms were nondose related	[120]

Isoniazid	INH-induced psychosis	8 cases of INH-induced psychosis out of 4960 hospitalised patients of pulmonary tuberculosis receiving INH	[121]

Isoniazid	Toxic psychosis as a psychiatric side effect during antituberculosis therapy occurred when isoniazid was given in dose ranging from 2.6 milligrams to 4.5 milligrams/kg bodyweight, over a period of eight to thirty six weeks.	Five cases developing psychosis while receiving isoniazid that presented with excessive argumentation, mental depression, euphoria, grandiose ideas, and complex delusions; none of these patients had any previous history of mental illness.	[122]

Ethambutol and isoniazid	Concomitant occurrence of INH- and EMB-induced psychosis in a single individual	A case report: an extremely uncommon event	[123]

Isoniazid	Symptoms of restlessness, irritability, emotional instability, agitation, apprehension, and fluctuation in behavior after isoniazid therapy	A case report	[124]

Isoniazid	A case of isoniazid psychosis in a 74-year-old, who developed restlessness, irritability, aimless activity, and incongruous actions 10 days after starting isoniazid therapy	A case report	[125]

Isoniazid	A case of isoniazid-induced psychosis with disturbed sleep, restlessness, and abnormal behavior	A case report	[126]

Ethambutol	A 40-year-old man with advanced HIV infection and mycobacterium avium complex infection experienced rapid cognitive decline after commencement of ethambutol, and symptoms fully resolved with cessation	A case report	[127]

Ethambutol	A case of a 51-year-old man with suspected tuberculosis (TB) pleurisy. An anti-TB trial with INH, rifampicin, and EMB was given initially. Dizziness, disorientation, and auditory and visual hallucinations developed after seven days of therapy. When the patient was challenged with EMB, the same psychiatric symptoms recurred but resolved again after discontinuation of EMB.	A case report	[128]

The neurological manifestations and toxicities of 12 antituberculosis drugs (isoniazid, rifampicin (rifampin), ethambutol, p-aminosalicylic acid, pyrazinamide, streptomycin, kanamycin, ethionamide, cycloserine, capreomycin, viomycin, and thiacetazone) are reviewed	In the Boston Collaborative Drug Surveillance Program performed in 1974. With rifampicin neurological complications have been observed infrequently, isoniazid is associated with a large number of accidental and intentional poisonings.	More than 35% of adverse effects associated with INH were psychiatric in nature, with an incidence of 1.9%. Adverse reactions to cycloserine are mainly dose related with neurological and psychiatric syndromes noted in up to 50% of patients. The highest incidence has been observed with Southwestern American Indians in which this agent was involved in 7% of all suicide attempts and 19% of the suicide deaths.	[129]

Isoniazid	In Peru, severe psychiatric syndromes associated with INH	Occurred in approximately 1.0% of tuberculosis cases between 1991 and 1999	[130]

Isoniazid	All case reports describing isoniazid-associated psychosis were reviewed. Studies were evaluated for the use of isoniazid, symptoms of psychosis, onset of symptoms, and dosage of isoniazid. The most common psychiatric symptoms associated with INH were delusions, generally presenting after approximately 4 weeks of taking the drug, and among patients of an average age of 35 years (range 17–53). They summarize risk factors as receiving a dose above 5 mg/kg; age 50 years or older; comorbid disease including diabetes mellitus, hepatic insufficiency, alcoholism, and hyperthyroidism; and past psychiatric history.	The incidence of isoniazid-associated psychosis is rare	[131]

Isoniazid	A patient who developed a psychotic disorder after 4 months of isoniazid prophylaxis for a positive tuberculosis tine test. His symptoms resolved within 2 weeks of discontinuing the isoniazid.		[132]

Primary antituberculosis (anti-TB) drugs	Out of 1149 patients with established tuberculosis who initially received anti-TB therapy neuropsychiatric manifestations were observed during the initial phase of therapy.	In 0.7% of TB patients	[133]

Ethionamide	Adverse reactions like anxiety, depression, and psychosis	Has been reported in 1%-2% of patients taking shorter courses of the drug, with higher rates reported with prolonged treatment	[134]

Ethionamide	A patient being treated with streptomycin, isoniazid, pyrazinamide, ethionamide, and prednisolone developed an acute psychotic reaction and died after jumping from a second floor window. It is probable that the reaction was precipitated by the ethionamide.	A case report	[135]

Ethambutol	Dizziness, disorientation, and auditory and visual hallucinations developed after seven days of therapy. Following discontinuation of anti-TB agents, the psychiatric symptoms subsided. When the patient was challenged with EMB, the same psychiatric symptoms recurred, but resolved again after discontinuation of EMB. EMB may be associated with mania, confusion, and psychosis.	A case report	[136]

Fluoroquinolones	Have been implicated in rare occurrences of psychosis, depression, suicidal ideation, delirium, and nightmares. CNS toxicity occurs in 1%–4.4% of patients but with serious adverse occurring in less than 0.5% of patients.	A case of a woman who experienced an acute psychosis secondary to ciprofloxacin administration, which resolved on cessation of therapy. Two cases of organic psychosis, induced by ofloxacin. 8861 patients receiving ciprofloxacin were assessed worldwide, and 138 cases presented with various neurological adverse reactions.	[137] [138] [139] [140]

Ofloxacin or ciprofloxacin	In a retrospective study conducted by the authors, 4189 reports of consultant psychiatric examinations were analyzed. In 29 patients, the suspicion of psychopathological ADR during treatment with ofloxacin or ciprofloxacin was documented. Psychopathological findings included delirious states, paranoid, depressive and manic syndromes, agitation, sleep disturbances, and stupor. In elderly patients, delirious and paranoid syndromes were predominant, whereas affective disturbances occurred more often in younger patients.	Reported psychiatric disturbance in 0.7% of 4189 individuals treated with either ofloxacin or ciprofloxacin.	[141]

Cycloserine	Severe psychiatric manifestations—including hallucinations, anxiety, depression, euphoria, behavioral disorders, and suicidal ideation and/or attempts. Psychiatric symptoms appear most likely to present within the first 3 months of treatment.	Have been reported to occur in 9.7%–50% of individuals receiving CS.	[142] [143] [144] [145] [146] [147] [148]

**Table 6:** Clinically significant drug-drug interactions involving TB medications and psychiatric medications.

TB medications	Psychiatric medications	Interactions	References
Isoniazid, in therapeutic dose		Was found to inhibit markedly plasma, but not platelet, MAO.	[149]

Drug that inhibits monoamine oxidase	SSRIs or TCAs.	Is contraindicated because of the potential to induce serotonin syndrome	[150]
		No reports of serotonin syndrome induced by combining SSRIs and isoniazid are published.	[151]

Isoniazid	Phenytoin and carbamazepine selected benzodiazepines (valium and others) Valproate Haldol.	Isoniazid can cause increased phenytoin and carbamazepine serum concentrations and toxicity. Isoniazid inhibits metabolism of selected benzodiazepines. Inhibition of monoamine oxidase and histaminase by Isoniazid can cause significant drug-food interactions. isoniazid has a biphasic effect of inhibition-induction on one cytochrome P450 isozyme, CYP2E1 and increases hepatic and CNS valproate toxicity and haldol toxicity.	[152] [153]

Isoniazid	2 patients who received isoniazid in conjunction with antidepressants. The first patient was prescribed sertraline (150 mg/day) in combination with isoniazid (300 mg/day). The second patient received nefazodone (400 mg/day) and buspirone (10 mg/day) in conjunction with isoniazid (300 mg/day).	None of patients reported adverse effects.	[150]

Isoniazid	SSRIs (citalopram, fluoxetine, fluvoxamine, paroxetine, and sertraline).	Are metabolized by similar mechanisms. Hepatic cytochrome P450 (CYP) enzymes are largely responsible for metabolism of isoniazid, citalopram, fluoxetine, fluvoxamine, paroxetine, and sertraline. While it has not been definitively established which isoenzymes are implicated in the metabolism of isoniazid, CYP2E1, CYP1A2, CYP2C9, CYP2C19, and CYP3A are inhibited to varying degrees by isoniazid. CYP2C19 and CYP3A were inhibited potently by isoniazid in a concentration-dependent manner. Both enzymes were inhibited approximately 40% by doses in the therapeutic range. Isoniazid induced competitive inhibition of CYP2D6 and weak noncompetitive inhibition of CYP2E1.	[154]

Isoniazid	Paroxetine is metabolized primarily by CYP2D6.	CYP2D6 is affected negligibly by isoniazid. The potential for drug interactions would appear to be minimal.	[24]

Isoniazid	Citalopram appears to be metabolized primarily by CYP2C19 and/or CYP3A4.	CYP2C19 and/or CYP3A4 are inhibited by isoniazid. It might not be the best choice for a patient taking isoniazid.	[155–157]

Isoniazid	Fluvoxamine is known to inhibit CYP1A2, CYP2C19, and possibly CYP3A3/4.	CYP1A2, CYP2C19, and CYP3A3/4 are inhibited by isoniazid	[158–160]

Isoniazid	Fluoxetine inhibits CYP2D6 and probably CYP2C9/10 significantly, and CYP3A3/4 and CYP2C19 to a lesser extent metabolite of fluoxetine, norfluoxetine inhibits CYP3A3/4 and has a half-life of 7 to 15 days.	CYP3A3/4 is inhibited by isoniazid increased potential for drug interactions.	[161–163]

Isoniazid	Sertraline probably inhibits CYP3A.	CYP3A is implicated in the metabolism of isoniazid.	[161, 162]

Rifampin	Antidepressants, haldol, quetiapine, methadone, phenytoin, valproic acid, lamotrigine, buspirone, benzodiazepines (diazepam, tiazolam), and zolpidem.	Reduces their levels.	[164]

Rifampin	Nortriptyline	A case report Higher than expected doses of nortriptyline were required to obtain a therapeutic drug level while the patient was receiving rifampin.	[165]

**Table 7:** Studies addressing factors affecting treatment adherence in TB patients.

First author/ references	Factors	Proposals
Pablos-Méndez, 1997 [166]	Of the 184, 48% patients were nonadherent. In multivariate analysis, only injection drug was used and homelessness predicted nonadherence.	These data lend support to directly observed therapy in tuberculosis.

Oscherwitz, 1997 [167]	46% of persistently nonadherent patients were homeless, 81% had drug or alcohol abuse, and 28% had mental illness.	Further improvements in the care of persistently nonadherent patients may require more psychosocial services, appropriate facilities for civil detention, and detaining patients long enough to assure completion of treatment.

Burman, 1997 [168]	18% who received outpatient DOT fulfilled one or more criteria for noncompliance. Risk factors for noncompliance were alcohol abuse and homelessness.	Innovative programs are needed to deal with alcoholism and homelessness in patients with tuberculosis.

Erhabor, 2000 [169]	The rate of compliance with antituberculosis regimen under directly observed therapy was found to be high (73%). DOT improves the rate of compliance. The only factor that significantly influenced rate of compliance was proximity to the chest clinic. Also, psychopathology could have adversely affected the rate of compliance.	Locating chest units in the existing primary health care facilities will improve the rate of compliance with antituberculosis therapy. More attention should be paid to behavioral aspect of tuberculosis control. Health workers involved in the management of these patients should develop a higher index of suspicion for possible psychopathology.

Manoharam (2001) [74]	66.7% of subjects completed their treatment. Only smoking was found to be associated with poor compliance in univariate analysis	The habit of smoking, disregarding own health, and not adhering to treatment instructions may be a reflection of the subject's personality.

Felton, 2005 [170]	Factors associated with adherence to treatment: patient related factors, provider characteristics, clinic facilities, characteristics of treatment regimens, and disease characteristics.	Adherence to treatment for latent tuberculosis infection: a manual for healthcare providers

Lavigne, 2006 [171]	Smoking prevalence was 21%. 72% of patients were adherent to LTBI treatment	Males and smokers need to have extra supervision to ensure compliance with LTBI treatment.

Naidoo, 2009[172]	Factors impacting adherence include: social and economic recourses prior to the onset and during the course of the disease, the causal attributions assigned to TB, the social, cultural, economic, disease related, and psychological challenges faced as a consequence of having TB, quality of health care received, use of traditional healing systems and feelings of helplessness, depression, and lack of social support.	Advocate a more holistic approach to health care programs with the inclusion of mental health services.

Munro, 2007 [37]	Structural factors: poverty, gender, and discrimination. Patient factors: motivation, knowledge, beliefs, and attitudes and interpretations of illness and wellness. Social context. health care service factors.	More patient-centred interventions, and far greater attention to structural barriers, are needed to improve treatment adherence and reduce the global disease burden attributable to TB.

Gelmanova, 2007 [303]	Substance abuse was identified as the only factor that was strongly associated with nonadherence.	Few TB programmes that have explicitly offered patients treatment for substance abuse generally have demonstrated better outcomes than “unexpanded” DOTS programmes.

K. Ito, 2008 [163]	Factors were classified into 7 categories; factors related to disbelief and/or prejudice for diagnosis and/or treatment (except factors related to drug adverse effects) were observed in 51.8%, factors related to economical problem in 24.1%, factors related to job or studies in 23.4%, factors related to drug adverse effects in 22.6%, factors related to visiting out-patients departments in 6.6%, psychiatric disease and/or drug abuse in 4.4%, others in 9.5%.	To improve the quality of tuberculosis medical care and services including good and sufficient explanations on TB and how to cure it and proper managements for drug adverse effects and then to expand public economical support for the costs of medicine and travel expenses to medical facilities and to make accessible time and place of the tuberculosis outpatient clinic more convenient and flexible for patients.

Norgbe, 2008 [173]	The factors contributing to noncompliance can be grouped into three categories, namely, patient related, health care, and community and treatment factors.	Develop and implement patient-centred interventions that encourage shared decision-making regarding treatment. Provide ongoing (in-service) training to health staff to improve and upgrade their competencies with regard to health education and communication skills. Strengthen patient support and community advocacy programmes aimed at eradicating the stigma associated with the disease. Emphasise the particular needs of individual patients and tailor the role of support systems to their needs. Plan interventions to reduce the influence of poverty and gender on patients and their treatment adherence

Husain, 2008 [88]	Depression and lack of perceived control over illness in those suffering from tuberculosis are reported to be independent predictors of poor adherence	Treating psychological problems in patients with tuberculosis may substantially improve treatment adherence.

Kruk, 2008 [174]	The majority of defaulters across the studies completed the 2-month intensive phase of treatment.	New TB chemotherapeutic agents which can reduce the length of treatment have the potential to improve global TB treatment success rates.

Matebesi, [175]	Lack of knowledge about TB, nonsustainability of educational campaigns, side effects of drugs, hunger and lack of family support, stigma attached to TB, and health-related factors such as the attitude of health care providers and the long delay in obtaining a diagnosis.	Recommendations are made for the instigation of enhanced education programmes focusing on patients, the community, and health care providers.

Bagchi, 2010 [176]	16% of patients among patients receiving DOTS treatment were nonadherent to the anti-TB therapy. Smoking during treatment and travel-related cost factors were significantly associated with nonadherence in the newly diagnosed patients, while alcohol consumption and shortage of drugs were significant in the residual groups.	Targeting easier access to drugs, an ensured drug supply, effective solutions for travel-related concerns, and modification of smoking and alcohol-related behaviors are essential for treatment adherence.

Kizub, 2012 [177]	Factors related to the patient (lack of means, being a migrant worker, distance to treatment site, poor understanding of treatment, drug use, and mental illness), medical team (high patient load, low motivation, and lack of resources for tracking defaulters), treatment organization (poor communication between treatment sites, no systematic strategy for patient education or tracking, and incomplete record keeping), and health care system and society.	Interventions to enhance TB treatment completion should take into account the local context and multilevel factors that contribute to default. Qualitative studies involving health care workers directly involved in TB care can be powerful tools to identify contributing factors and define strategies to help reduce treatment default.

Yin, 2012 [178]	Nine factors conceptually associated with medication adherence in TB patients: (1) communication with healthcare providers, (2) personal traits, (3) confidence in curing TB, (4) social support, (5) mood disorders, (6) lifestyle and habits, (7) coping style, (8) access to healthcare, and (9) forgetfulness.	A 30-item TB medication adherence scale (TBMAS) with a positive predictive value of 65.5% and sensitivity of 82.9% was developed and incorporated the latest research in TB specific medication adherence, where predictors for adherence such as patient behavior and patient-provider interaction in TB treatment have been explored. The resulting tool will help TB medical professionals identify not only TB patients with poor adherence but also potential reasons for nonadherence and help them to design and implement targeted interventions to improve adherence.
